# Chloroquine, an autophagy inhibitor, potentiates the radiosensitivity of glioma initiating cells by inhibiting autophagy and activating apoptosis

**DOI:** 10.1186/s12883-016-0700-6

**Published:** 2016-09-20

**Authors:** Hongxing Ye, Mantao Chen, Fei Cao, Hongguang Huang, Renya Zhan, Xiujue Zheng

**Affiliations:** Department of Neurosurgery, First Affiliated Hospital, School of Medicine, Zhejiang University, No. 79 Qingchun Road, Hangzhou, Zhejiang People’s Republic of China

**Keywords:** Glioma initiating cells, Radiosensitivity, Irradiation, Apoptosis, Autophagy, Chloroquine

## Abstract

**Background:**

Glioblastoma is refractory to conventional treatment, which is combined of surgery, chemotherapy and radiotherapy. Recent studies have shown that glioma initiating cells (GICs) contribute to tumorigenesis and radioresistance. Recently, other studies showed that the GICs use the autophagy as the major pathway to survive. Chloroquine, an anti-malarial chemical, is an autophagic inhibitor which blocks autophagosome fusion with lysosome and slows down lysosomal acidification. The aim of this study was to explore the mechanisms of chloroquine on the radiosensitivity of GICs.

**Methods:**

Human glioblastoma cell lines U87 were investigated. MTT and clonogenic survival assay were used to evaluate the cell viability and survival from radiation. The formation of autophagosomes were evaluated by immunofluorescence. Annexin V-FITC/PI staining and flow cytometry were used to quantify the apoptotic cells. The expression levels of proteins were analyzed by Western blot. Cell cycle status was analyzed by checking DNA content after staining with PI. A comet assay was used to assess the DNA repair in the cells. Tumorsphere assay was used for evaluating GICs’ renewal ability.

**Results:**

Treatment of U87 GICs with chloroquine (10–80 nmol/L) alone inhibited the cell growth in a dose-dependent manner. A dose of chloroquine (20 nmol/L) obviously enhanced the radiation sensitivity of U87 GICs., we found more punctate patterns of microtubule-associated protein LC3 immunoreactivity in radiation-treated U87 GICs, and the level of membrane-bound LC3-II was obviously enhanced. A combination of radiation and chloroquine obviously enhanced the U87 GICs’ apoptosis, as demonstrated by the enhanced levels of caspase-3, and reduced level of Bcl-2. In additon, combination of radiation and chloroquine cause G1/G0 cell cycle arrest. what’s more, Chloroquine obviously weakened the repair of radiation-induced DNA damage as reflected by the tail length of the comet. Combination treatment of irradiation and chloroquine has synergistic effects on decreasing the GICs’ tumorsphere number and diameter.

**Conclusion:**

Chloroquine enhances the radiosensitivity of GICs in vitro, suggesting the feasibility of joint treatment with chloroquine with radiation for GBM.

## Background

Glioblastoma (GBM) is the most malignant and common primary neoplasm in central nervous system. Despite the advances in conventional treatments, comprised of surgical resection, radiotherapy and chemotherapy, the median duration of survival of GBM is 14.6 months after first diagnosis [1]. Recent studies report that a minority subset of the whole glioma population is glioma initiating cells (GICs), which led to tumorigenesis, because these GICs show enhanced self-renewal ability, multipotent differentiation that GICs can epitomize the original tumor in vivo [2]. GICs are considered “seed” cells and have strong radio-chemoresistance and tumorigenic potential [3, 4]. Bao S et al. found primary tumor cells are less radioresistant than GICs, and GICs were responsible for tumor recurrence after radiotherapy [5]. Although surgery followed by radiation and chemotherapy could eradicate most part of glioma cells, they did not kill GICs. Thus, GICs are the novel cellular targets and their elimination can hinder cancer progression and recurrence.

Autophagy is also named type II programmed cell death pathway, which is different from the apoptotic type I death pathway [6]. Autophagy is a balance that weights between reconstruction, energy equilibrium, cell death and survival, correspondingly, with the internal or external stimulations accepted. Autophagy has different effects on tumor outcomes: it is essential for some forms of cancer cell death, but it can help cancer growth by assisting cancer cells fight hard cancer environment (e.g., malnutrition, shortage of oxygen) and combat radiotherapy and chemotherapy [7]. Yao found that ionizing radiation (IR) of human glioma cells led to increased autophagy [8]. Other studies showed that the induction of autophagy by radiotherapy result in the radioresistance of GICs [9]. Therefore, discovery of a novel combination treatment, such as radiotherapy or chemotherapy combines autophagy suppression may be a feasible and promising strategy.

Chloroquine is an anti-malaria drug, which has been used for over eighty years. Recent years, choloroquine, as an autophagy inhibitor, is drawing more and more attentions [10]. Chloroquine-treated tumor cells are not able to exploit autophagy as an substituting source of energy and will die [11].

Researchers found that chloroquine combined with chemo-radio-therapy, increased the duration of survival of glioblastoma patients [12, 13]. However, other researchers reported that the chloroquine treatment was found not to be effective for the medical treatment of malignant astrocytomas [14].

Therefore, the clinical effect of joint treatment with radiation and chloroquine on the radioresistance of malignant glioma and GICs remains uncertain. We investigate the potential radiosensitization effect and its probable mechanisms of chloroquine on GICs.

## Methods

### Preparation of CD133^+^ U87 GICs, cell culture, and reagents

The human glioma cell lines U87 were purchased from the Shanghai Institute of Biochemistry (Shanghai, China). To isolate human CD133^+^ U87 GICs, Glioma cell line U87 was cultured at 37 °C in the presence of 5 % CO2 in the medium containing 90 % Dulbecco’s modified Eagle’s medium (DMEM) and 10 % Fetal Calf Serum (FBS). After having digested with trypsin, resuspended, centrifuged, and purified by magnetic separation using anti-CD133 microbeads (Miltenyi Biotec) per the manufacturer’s instructions, CD133 negative cells were also obtained at the same time and cultured in conditions appropriate for growth. The human CD133+ U87 GICs, were then cultured at 37 °C in the presence of 5 % CO2 in the serum-free DMEM/F12 culture medium, supplemented with B27 (2 %), firoblast growth factor, N2 supplement, and epidermal growth factor. The medium was replaced every 3–5 days. We observed and counted the attachment and suspension of cells twice per day. Chloroquine was purchased from Sigma.

### Cell viability assay

Cell viability was measured by MTT assays. Cells were seeded in 96-well microplates (1 × 10^5^ cells or 1 × 10^4^ cells/well). After incubation at 37 °C in the presence of 5 % CO2 in constant temperature and humidity incubator for 3–5 days, the tumor cells were treated using different doses of chloroquine or radiotherapy alone or in combination. We set up a control group and a zero adjustment group. At specified time points, 20ul of MTT solution (5 mg/mL) was added 4 h before the end of the incubation duration, the reaction was stopped by the addition of 150 μL dimethylsulfoxide (DMSO). The optical density (OD) or absorbance was read at 490 nm.

### X-rays

Radiation was performed using a 6-MV X-rays from a linear accelerator (PRIMUS, DE, Siemens A&D LD, Nelson Avenue Concord, USA).

### Clonogenic assay

Cells were seeded in six-well plates (2 × 102 cells/well). After 12 h incubation, cells were treated and irradiated. After two weeks, the colonies were fixed with methanol for 15 min and stained with 0.5 % crystal violet. Colonies with at least 50 cells were counted.

### Immunofluorescence

The U87 GICs were plated onto coverslips and treated with 20 μM/L concentration of chloroquine and 6 Gy dose of radiation for 24 h. After fixation with 4 % paraformaldehyde, tumor cells were incubated with blocking buffer (0.1 % Triton X-100, 2 % horse serum) for 30 min at 37 °C, and with the rabbit anti–human light chain 3 antibody (LC3) (1:200, Abcam) at 4 °C for overnight. After wash, a secondary antibody (1:200) conjugated to Alexa 488 (Invitrogen) was added and incubated for 1 h at room temperature. Fluorescent cells were examined with a confocal microscope to appraise the formation of autophagosomes.

### Western blot analysis

The cells were lysed with a lysis buffer (Beyotime, Haimen, China), and centrifuged at 12,000 × g for 20 min. The total protein concentration was determined using the a BCA protein assay kit (Pierce). Samples were resolved in SDS-PAGE, transferred to 0.45 μm nitrocellulose transfer membranes and analyzed separately. After blocking with 5 % skim milk at room temperature for 60 min, the blots were survey with primary antibodies against mouse anti-Bcl-2 (1:500; Abcam), rabbit anti-LC3 (1:1000; Abcam), mouse anti-GAPDH (1:1000; Cell Signal), and rabbit anti-active caspase-3 (1:1000; Abcam), at 4 °C for overnight. We washed the membranes three times with TBST buffer (20 mmol/L Tris-buffered saline and 0.1 % Tween 20) for 1 h. And we used peroxidase conjugated anti-mouse-IgG/anti rabbit-IgG as secondary antibodies. We did Chemiluminescence with Amersham ECL plus Western blotting detection system (GE healthcare). After washing with the TBST buffer, the membranes were measured with the Odyssey Infrared Imaging System (LI-COR).

### Flow cytometric apoptosis assay

The cells in the different treatment groups were measured using an Annexin V FITC Kit (Sigma, St Louis, MO). Earlier stages apoptotic (annexin V+) (PI-) cells were identified by flow cytometry on a BD FACS flow cytometer (San Diego, CA).

### Cell cycle analysis

5 × 10^5^ cells per sample were collected after centrifugation at 1000 r.p.m for 5 min. The cells re-suspended with 0.5 ml PBS and fixed with ice-cold 70 % ethanol overnight at 4 °C. Fixed cells were washed with PBS and stained with propidium at room temperature for 1 h. The cells were subsequently measured by FACScan (Becton-Dickinson, San Diego, CA, USA).

### DNA damage

A comet assay quantitatively assesses DNA repair in U87 GICs. The detailed procedure of the comet assay performed in this experiment refers to the report of Wang et al. [15]. The comet assay metric for DNA damage induced by the treatment was tail DNA (%). We analyzed images from 50 cells (25 from each replicate slide).

### Neurosphere assay

We trypsinized the U87 GICs and seeded equal numbers of GICs in 48 well plates, and treated as indicated. Five days after treatment, we counted total neurospheres numbers and photographed.

### Statistical analysis

The experimental data is showed as the mean ± SD. All experiments were performed three times. Unpaired Student’s *t* test was performed to analyses the significance between experimental groups. Statistically significant *P* values are indicated in the figures with asterisks: **, *P* < 0.01; *, *P* < 0.05. We used GraphPad software (Inc.; version 5.02) for all statistical analyses.

## Results

### Irradiation and chloroquine had synergetic effect on U87 GICs

**Fig. 1:**
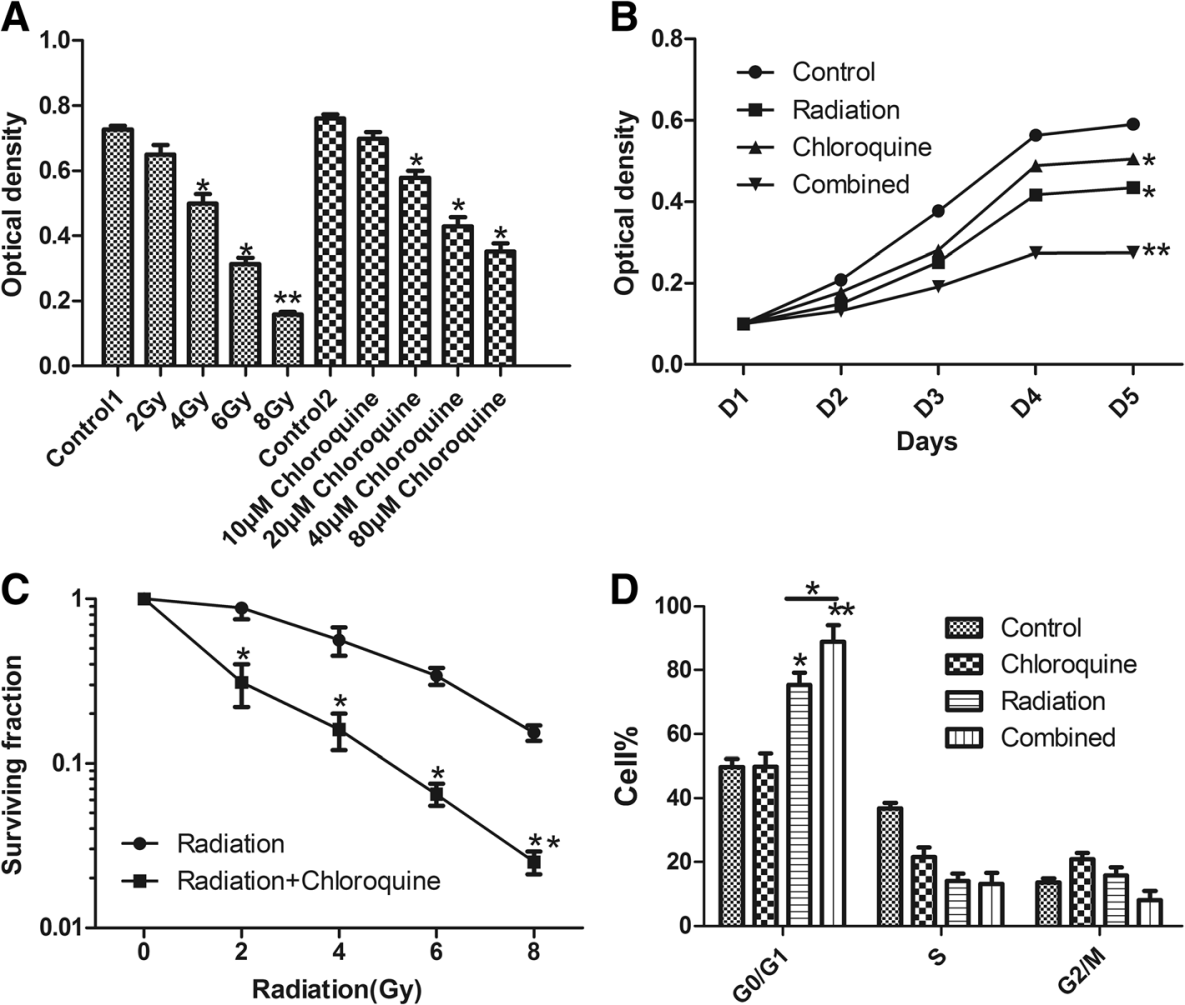
Effects of X-ray and chloroquine alone or combination on the viability of U87 GICs. **a** Human glioma cell line U87 GICs were plated in 96-well plates(1 × 105/well), treated with various dose of X-ray or different concentration of chloroquine alone for 72 h. Cell viability was examined by MTT assay. It demonstrated that X-ray and chloroquine suppressed cell viability in dose-dependent manner. Each bar represents mean ± SD of triplicate determinations; results shown are representative of three identical experiments. **b** U87 GIC were plated in 96-well plates(5 × 104/well), and treated with 6Gy X-ray or 20 μM chloroquine alone or combination. At indicated time points a MTT assay was performed to assess the cell number, as measured by absorbance(OD). **c** Clonogenic survival 14 days after treatment with radiation alone(0Gy to 8Gy), radiation plus chloroquine(20 μM). (*p < 0.05,**p < 0.05, *n* = 3). **d** Quantification of cell cycle distribution of U87 GIC, using flow cytometry and PI, staining 48 h after treatment. Radiation combined chloroquine significantly increased number in G0/G1 phase compared to radiotherapy alone group (*p < 0.05, **p < 0.05, *n* = 3)

The inhibitory effect of irradiation or chloroquine on cell viability in GICs was evaluated after 72 h treatment. As shown in Fig. 1a, a dose-dependent inhibitory action on cell growing was assessed with irradiation or chloroquine alone. As shown in Fig. 1b, we seeded cells onto 96-well plates, and quantified the number of cells at indicated time points by MTT assay. Irradition and chloroquine had synergistic inhibitory effect on U87 GICs. Furthermore, We found that clonogenicity of U87 GICs treated with radiation or radiation plus chloroquine were obviously decreased in an radiation-dose dependent manner (Fig. 1c). And the clonogenicity of the combined group was significantly decreased, compared with radiation treatment alone, suggesting chloroquine treatment obviously enhanced radiation sensitivity in GICs. As shown in Fig. 1d, there was obviously enhanced in the number of cells in the G0/G1 phase in the combined group (88.91 % ± 5.13 %) compared with the control group (49.75 % ± 2.58 %). This increase suggested that combination treatment of irradition and chloroquine significantly promoted a cell-cycle arrest in G0/G1 phase.

### IR with chloroquine synergistically induced apoptosis

**Fig. 2:**
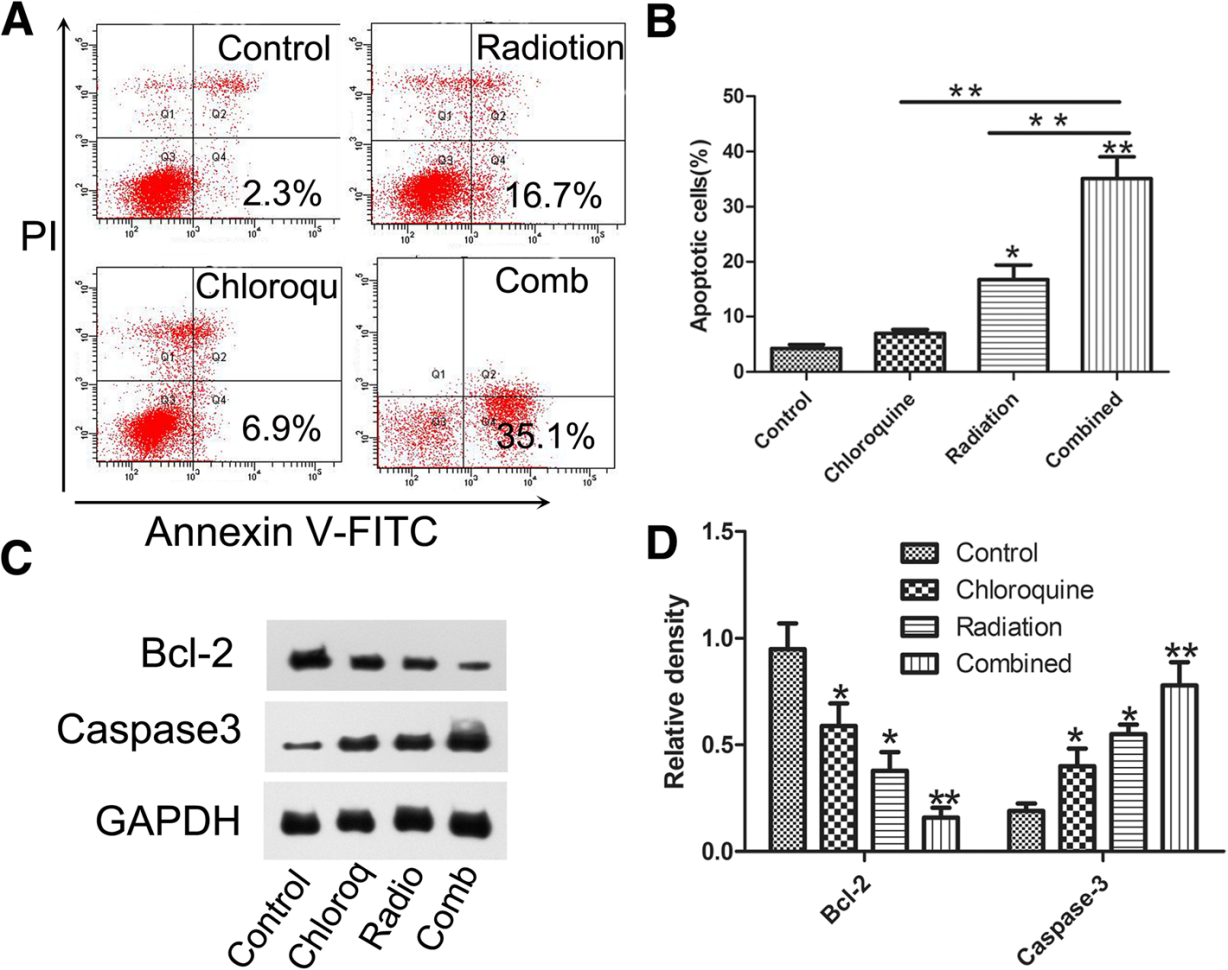
Combination treatment of irradiation and chloroquine obviously promoted apoptosis in U87 GIC. **a** U87 GIC were treated with X-ray(6Gy) alone and chloroquine(20 μM) alone or in combination for 24 h, and the promotion of apoptosis was measured using AnnexinV-FITC/PI double staining. **b** Quantitative analysis of apoptotic cells. Data are demonstrated as the mean ± SD,*, *p* < 0.05, **p < 0.01, *n* = 3. **c** Apoptosis -associated proteins were investigated by Western blotting. **d** Relative density of Bcl-2 and Cleaved caspase-3 versus GAPDH in each sample measured by densitometry of the blots. Densitometric analysis of the immunoblot is expressed as a percentage of control. Data are presented as the mean ± SD, *, *p* < 0.05 versus control, **p < 0.01, versus control, *n* = 3

To explore whether the synergistic cytotoxicity was related to apoptosis, U87 GICs were processed with X-ray(6Gy) or chloroquine(20 μM) alone or combination of both for 24 h, and the induction of apoptosis was assessed by AnnexinV-FITC/PI double staining. As shown in Fig. 2a-b, The percentage of early apoptotic tumor cells(AnnexinV-FITC positive, PI negative) in the combined treatment(35.06 % ± 6.98 %) was significantly higher than that with radiation (16.73 % ± 4.52 %) or chloroquine(6.94 % ± 1.21 %) alone.

To investigate the expression of proteins associated with apoptosis in U87 GICs, we cultured cells with 6Gy radiation or 20 μM chloroquine alone, or the two in combination for 24 h. We The analyzed the expression levels of apoptosis associated proteins from each group by Western blotting. Combined treatment obviously increased the expression of Caspase-3(Fig. 2c), and decreased the expression of the antiapoptotic Bcl-2.

### Chloroquine inhibits autophagy induced by IR

**Fig. 3:**
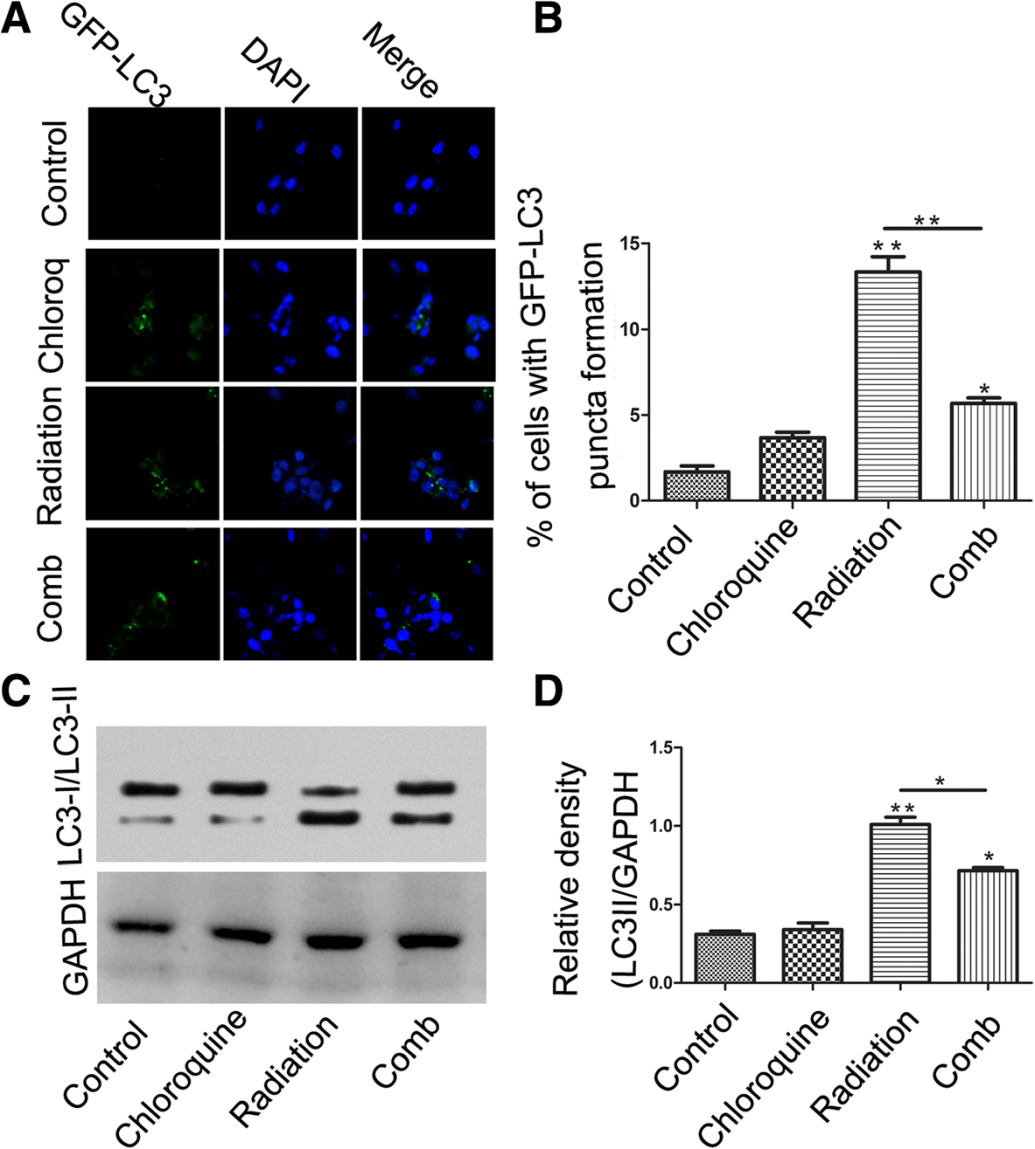
Irradiation obviously promoted autophagy and obviously enhanced autophagy associated proteins expression in U87 GICs, and chloroquine can significantly inhibit the autophagy process which induced by radiation. **a** Representative images(×200) of the indicated regions of U87 GICs, which analyzed for LC3 expression by immunofluorescence analysis. **b** Quantitative analysis of the number of cells with punctuate GFP-LC3. **c** Authophagy-associated protein LC3 level in U87 GICs were examined by Western blotting. **d** Quantification of LC3 protein expression in U87 GICs treated by indicated treatments after normalization with GAPDH levels. Mean ± SD. *n* = 3. (*, *p* < 0.05;**p < 0.01)

LC3-II could be seen in punctate structure mainly representing autophagosomes. The location of LC3 was assessed by indirect immunofluorescence microscopy. By fluorescence microscopy assay(Fig. 3a-b), the numbers of GICs with LC3+ puncta in the radiation alone group (13 %) was obviously more than that in chloroquine alone (4 %) or control (2 %) group, and in combined group (6 %), chloroquine significantly inhibited the autophagy induced by IR. Moreover, to test whether different treatments induced different autophagy in U87 GICs, we assessed the protein expression of LC3-II, a mammalian homologue of ATG8 located in autophagosomes. U87 GICs were treated with 6Gy IR or 20 μM chloroquine alone, or the combination of the two for 48 h, and the ratio of the LC3-II/LC3-I were determined by western blot. As shown in Fig. 3c-d, LC3-II level was obviously enhanced in radiation group, and it was significantly decreased by the combined treatment.

### Chloroquine weakens the repair of radiation-induced DNA damage in GICs

**Fig. 4:**
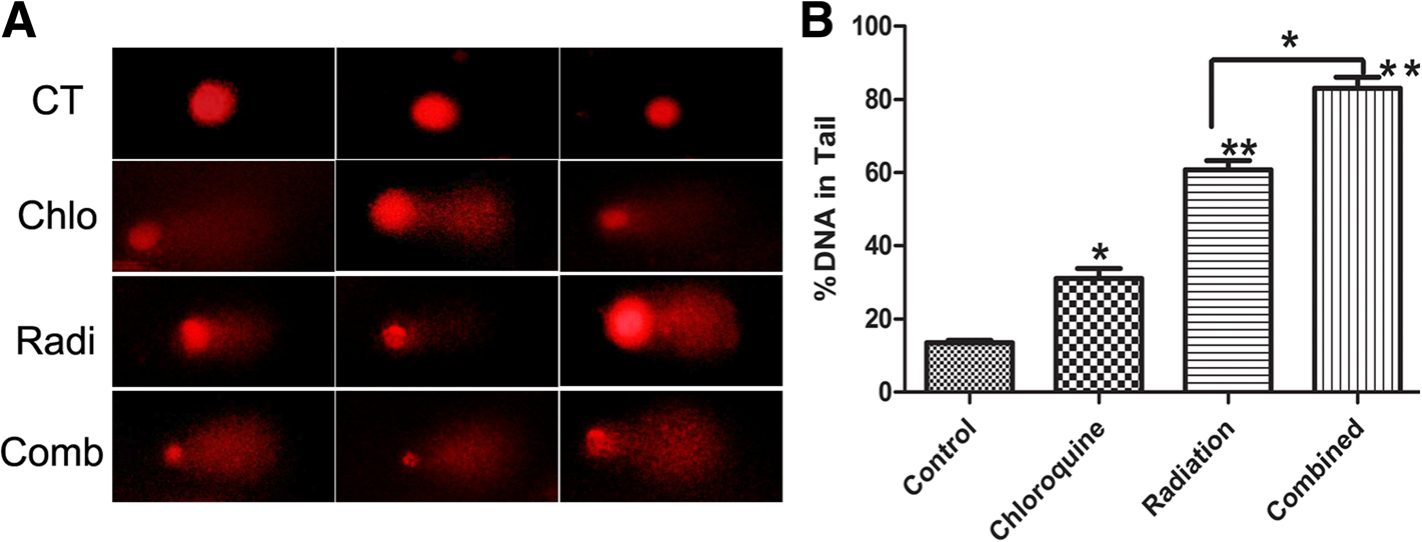
Chloroquine weakens the repair of IR-induced genomic DNA damage in GICs. **a** GICs were treated radiation(6Gy) and chloroquine(20 μM) and harvested at 24 h after radiation. The alkaline comet assay was used to measure treatment-induced DNA damage. Representative image of U87 GICs at 24 h after different treatments are demonstrated (800 × magnification). **b** The quantification of the percentage of cells with comet tails at 24 h after treatment

As one of pivotal mechanism involved in radioresistance is DNA repair, the effects of chloroquine on radiation-induced celluar genomic DNA damage were examined by the alkaline comet assay. In our study, we assessed genomic DNA damage in GICs at 24 h after treatment. We determined DNA damage by assessing the tail length of the comet with a microscope. DNA damage was more severe in the combined group than in IR or chloroquine alone group at 24 h after treatment. The data is demonstrated as the mean ± SD from more than 50 cells. Each group was analyzed in 3 times (Fig. 4b). Then, chloroquine treatment was used to prevent the repair of radiation-induced DNA damage.

### Co-treatment inhibits self -renewal effect on U87 GICs

**Fig. 5:**
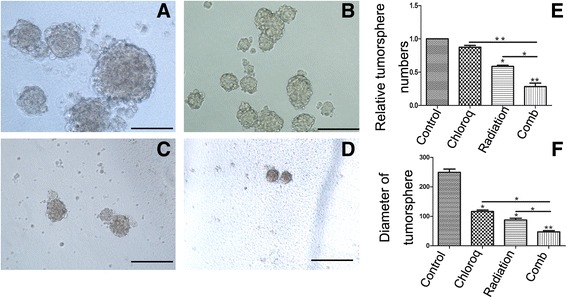
Combination treatment of irradiation and chloroquine have synergistically effects on decreasing the GICs’ tumorsphere numbers and diameters. **a-d** U87 GICs were treated as described before, then seeded in serum-free medium to generate tumorspheres,5 days after treatment, we counted and photographed total tumorspheres (**a **control group, **b** chloroquine group, **c** radiation group, **d** conbination group). **e** Quantitative analysis of the relative tumorsphere numbers treated by indicated treatments. **f** Quantitative analysis of the diameter of the tumorspheres treated by indicated treatments. (*, *p* < 0.05, **p < 0.01)

Sphere forming assay has been widely applied to monitor the self-renewal ability of GICs. We observed the combined treatment had strong inhibitory effects in tumorsphere forming assays conducted as a stem cell surrogate assay to measure the effect on clonogenic cells. In this study, the combination of chloroquine(20 μM) and a 6Gy ionizing radiation turned out to be very potent, probably because of the strong cytostatic effect on GICs. As shown in Fig. 5, the number of the tumorsphere in the combined treatment was significantly smaller than that in chloroquine or IR alone group. The diameter of tumorsphere (47 ± 8 μm) in the combination was also smaller than that in chloroquine (116 ± 9 μm) or IR (87 ± 11 μm) alone group.

## Discussion

In the present study, we explored the radiosensitizing action of an autophagy blocker chloroquine in GICs, and aimed to further understand the molecular mechanisms of its effect. We found that chloroquine or radiation alone inhibited U87 GICs growth in a dose-dependent manner. The combined inhibitory effect of chloroquine and radiation on the viability of human GICs was stronger than that of chloroquine or radiation alone, in a synergetic way (Fig. 1a-c). The response of GICs to radiation was associated with autophagy and the late autophagy inhibitor chloroquine in combination with radiation caused G0/G1 phase arrest, promoted GICs’ apoptosis and impaired the repair of radiation-induced DNA damage in GICs, and decreased the GICs’ tumorsphere numbers and diameters.

Recent researches demonstrated that cancer stem cells could exploit autophagy to survive and accelerate their renewal [16, 17]. GICs, as the most radioresistant cell subset and autophagy targeting, may be helpful to cure the disease [18, 19]. Our data indicates that irradiation of U87 GICs results in enhanced autophagy. Chloroquine in combination with radiation inhibited the autophagy induced by radiation and strongly promoted GICs apoptosis. Apoptosis involved many diseases, especially malignant tumors. There are two main apoptotic pathways: the extrinsic or death receptor pathway and intrinsic or mitochondrial pathways [20]. Study finds that radiation is a stimulus that initiate the intrinsic pathway. The control and regulation of intrinsic pathway is through members of Bcl-2 family of proteins [21]. Here, we found that the combination of chloroquine and radiation reduced anti-apoptotic Bcl-2, up-regulated caspase-3, and enhanced the fraction of Annexin V-FITC positive and PI negative cells to 35.1 %. However, about 16.7 and 6.9 % respectively were measured with treatment of radiation or chloroquine alone (Fig. 2). These results may suggest that the combined inhibitory effect cause apoptosis at least in part by the intrinsic pathway. These results demonstrate that chloroquine plays a synergetic role in radiation-induced viability inhibition and apoptosis in U87 MG GICs. The result may be helpful to explaining the results of a clinical trial that chloroquine, added to a comprehensive therapeutic protocol (operation plus radiotherapy and chemotherapy) for glioblastoma, significantly prolonged the median overall survival compared with control groups [22]. Other study on the combining of radiation with chloroquine for the management of recurrent glioblastoma validated the feasibility of the regimen and the authors reported encouraging therapy outcomes [23].

Growing evidences suggest that chloroquine is a strong anticancer drug in managing several cancers, such as leukemia and hepatocarcinoma [24, 25]. Yuan et al. showed that suppression of ATG5 by siRNA or suppression of autophagy using 3-methylademine enhanced the radiosensitisation action of gliomas after STAT3 suppression [26]. Other research demonstrated that high expression levels of early growth response 1, which induces autophagy, is produced by the resistant clones of GICs [27]. In addition, mitochondrial isoenzyme of NADP + −dependent isocitrate dehydrogenase siRNA-transfected A172 glioma cells were sensitised after suppression of autophagy [28]. Firat, E et al. also demonstrated that induced-autophagy effects of PI3K/Akt pathway inhibitors obviously prevent cell death induction in γ-irradiated GICs, however, chloroquine significantly promotes γIR-induced cell death in highly radioresistant GICs [29]. Our study should be of high clinical significance, one reason is all data presented are focused on GICs, a population thought to be pivotal, because for cancer progression and therapy resistance that must be uproot in order to obtain long-term recurrence-free survival, another reason is that a clinically applicable late autophagy inhibitor was used. However, the present study has two limitations. First, our data presented here have been obtained from cell lines, not from patients’s tumor-derived stem-like cells. Second, the further experimentation with animal models and clinical trials were not investigated in this study.

## Conclusion

We have shown that inhibition of autophagy in combination with radiation is a promising therapeutic strategy for targeting GICs. The radiosensitization efficiency of chloroquine is obtained by inhibiting autophagy, weakening the capacity for DNA repair in the early stage and promoting cell-cycle arrest in addition to apoptotic responses.

## Abbreviations

DMSO: Dimethylsulfoxide
EDTA: Na2-ethylenediaminetetraacetic acid
GBM: Glioblastoma
GICs: Glioma initiating cells
IR: Ionizing radiation
LC3: Light chain 3
OD: Optical density
PBS: Phosphate-buffered saline
PI: Propidium iodide
